# Ultrahigh brilliance quasi-monochromatic MeV γ-rays based on self-synchronized all-optical Compton scattering

**DOI:** 10.1038/srep29518

**Published:** 2016-07-13

**Authors:** Changhai Yu, Rong Qi, Wentao Wang, Jiansheng Liu, Wentao Li, Cheng Wang, Zhijun Zhang, Jiaqi Liu, Zhiyong Qin, Ming Fang, Ke Feng, Ying Wu, Ye Tian, Yi Xu, Fenxiang Wu, Yuxin Leng, Xiufeng Weng, Jihu Wang, Fuli Wei, Yicheng Yi, Zhaohui Song, Ruxin Li, Zhizhan Xu

**Affiliations:** 1 State Key Laboratory of High Field Laser Physics, Shanghai Institute of Optics and Fine Mechanics, Chinese Academy of Sciences, Shanghai 201800, China; 2 IFSA Collaborative Innovation Center, Shanghai Jiao Tong University, Shanghai 200240, China; 3 State Key Laboratory of Intense Pulsed Radiation Simulation and Effect, Northwest Institute of Nuclear Technology, Xi’an 710024, China

## Abstract

Inverse Compton scattering between ultra-relativistic electrons and an intense laser field has been proposed as a major route to generate compact high-brightness and high-energy γ-rays. Attributed to the inherent synchronization mechanism, an all-optical Compton scattering γ-ray source, using one laser to both accelerate electrons and scatter via the reflection of a plasma mirror, has been demonstrated in proof-of-principle experiments to produce a x-ray source near 100 keV. Here, by designing a cascaded laser wakefield accelerator to generate high-quality monoenergetic e-beams, which are bound to head-on collide with the intense driving laser pulse via the reflection of a 20-um-thick Ti foil, we produce tunable quasi-monochromatic MeV γ-rays (33% full-width at half-maximum) with a peak brilliance of ~3 × 10^22^ photons s^−1^ mm^−2^ mrad^−2^ 0.1% BW at 1 MeV. To the best of our knowledge, it is one order of magnitude higher than ever reported value of its kinds in MeV regime. This compact ultrahigh brilliance γ-ray source may provide applications in nuclear resonance fluorescence, x-ray radiology and ultrafast pump-probe nondestructive inspection.

Laser wakefield accelerators^1^ (LWFA) have achieved significant progress recently owing to the sophisticated injections, cascade and guiding technologies^2^,^3^,^4^,^5^,^6^,^7^,^8^, and they can produce monoenergetic, energy tunable, GeV-class femtosecond electron beams (e-beams) with tens of pC charge over a distance of centimeter-scale^6^,^8^,^9^,^10^,^11^,^12^,^13^,^14^, which hold the potential of becoming compact alternatives to conventional radio-frequency-based linear accelerators^15^. The properties of the LWFA e-beams qualify them as a unique driver for the generation of well-collimated, near-monochromatic, tunable, ultra-short and high peak brilliant x- and γ-ray sources^16^. Wiggling of the LWFA e-beams either in the conventional periodical magnetic field structures (undulator radiation)^17^, strongly focusing laser wake-fields (betatron radiation)^18^, or intense laser fields (Compton scattering)^19^,^20^,^21^,^22^,^23^,^24^,^25^ have recently been experimentally realized to emit high-energy photons. Of these schemes, the Compton scattering using LWFA e-beams, which has been successfully demonstrated to increase γ-rays’ brilliance by 10000 folds over that from the conventional accelerators, offers the most promising route to generate compact bright hard x- or γ-ray sources up to a few MeV, and will enable many practical applications.

All-optical Compton scattering based on two laser pulses, the first to drive the plasma-accelerator and the second to collide with the accelerated e-beam, has been demonstrated to produce MeV γ-rays with divergence of ~10 mrad, total photons of ~10^7^, but had large bandwidths due to the large-energy-spread e-beams^21^. By improving the energy spread of the laser-accelerated e-beams to ~25%, the x-rays bandwidth was reduced to 50% at 66 keV^22^. While increasing the scattered laser intensity i.e. *a*_s_ > 2, multi-MeV γ-rays where harmonics dominated the high-energy region due to the strong nonlinear effects have also been produced with continuous spectra^23^. Nowadays this head-on collision scattering scheme using two laser pulses is still an experimentally challenging task, which requires complicated control of two 100 TW-class laser pulses to ensure the overlap in time and space between the e-beam and the scattered laser pulse. To avoid alignment and synchronization issues of the two-laser scheme, another smart and robust scheme, which employs just a laser pulse to both accelerate electrons and scatter via the reflection of a plasma mirror^20^,^24^, can guarantee the overlap both in time and space inherently and has been firstly demonstrated in proof-of-principle experiments to produce 100 keV x-rays^20^. However, the produced x-rays had continuous spectra due to the broadband e-beams, containing non-negligible bremsstrahlung radiation as well. Similar to this setup, an study^24^ was performed that this self-aligned Compton scattering could generate 75~200 keV x-rays with 50% FWHM energy spread. To further achieve the desired MeV-ray sources with narrower bandwidth and higher peak brilliance, higher energy laser-accelerated e-beams as well as better qualities such as lower energy spread, larger charge and smaller emittance are essential.

In this article, we report the generation of quasi-monochromatic MeV γ-rays by using a self-synchronized all-optical Compton scattering scheme. Based on our previous work^6^,^8^,^26^,^27^, a novel cascaded LWFA is designed here to generate high-quality e-beams (~1% rms energy spread, ~50 pC at the peak energy, tunable from 150 to 450 MeV, <0.5 mrad rms divergence). Then the e-beams collide with the intense driving laser pulse via the reflection of a thin foil, either 20-um-thick titanium (Ti) or 30-um-thick aluminum (Al), which is fixed on a movable mount and positioned close to the right exit of the gas jet as a plasma mirror^28^ (the reflectivity is close to 70% in our PIC simulations at *a*_0_ ≈ 1). Based on this scheme, the back-reflected laser pulse with intensity of *a*_0_ ≈ 1 at collision point would be beneficial to improve the photon yield up to ~5 × 10^7^ per shot and at the same time avoided a remarkable nonlinear bandwidth broadening. Quasi-monochromatic γ-rays tunable from 0.3 to 2 MeV with a bandwidth of ~33% (FWHM) and a small divergence of 4 mrad have been achieved. This photon source has an ultrahigh peak brilliance of ~3 × 10^22^ photons s^−1^ mm^−2^ mrad^−2^ 0.1% BW. It can provide unique properties that may be of interest for a few practical applications such as ultrafast x-ray or γ-ray radiology and photonuclear research in the future.

## Results

### High-quality LWFA e-beams generation

**Figure 1:**
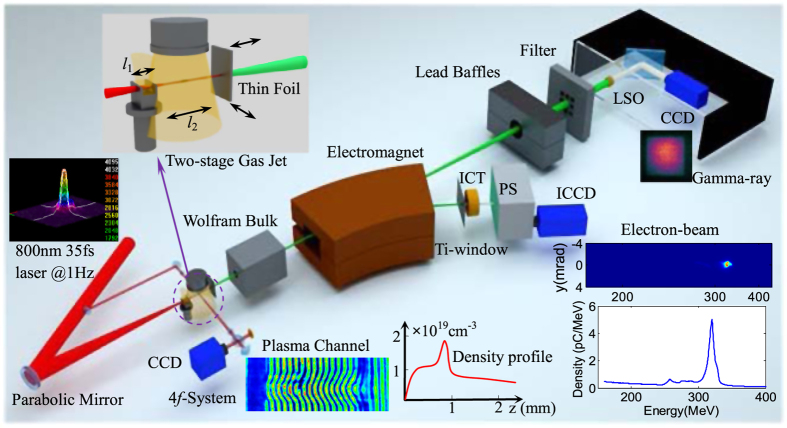
Layout of the self-synchronized All-optical Compton Scattering Experimental setup. The 0.8-um-wavelength, 33*f*s-duration pulses from the 200 TW Ti:sappire laser system were focused onto the two-segment (0.8 + 3 mm) pure helium gas jets onto the supersonic nozzles. About 5% of the pump laser was split off as a probe pulse, which crossed the interaction region perpendicularly into a 4*f* Michelson-type interferometer system, to measure the plasma density profile and monitor the laser evolution. The produced e-beams were deflected by a 90-cm-long dipole electromagnet with a maximum magnetic field of 1.1 Tesla, and measured by a Lanex phosphor screen (PS) imaged onto an intensified charge-couple device 16-bit (ICCD) in a single shot. A 30-um-thick Al foil or 20-um-thick Ti as a plasma mirror was presented to reflect the driving laser to scatter the generated e-beams to produce γ-rays. A 10-cm-thick wolfram collimator with a 2-mm hole was placed to block the irrelevant radiation. The lead-grid filter and Lu_2_SiO_4_(LSO)-crystal scintillator with a diameter of 50 mm were placed 5.2 m downstream, imaged with a CCD camera encased by the lead baffles for detecting γ-rays.

Figure 1 shows a schematic layout of the experimental setup. The 33*-f*s, 800-nm laser pulses^29^ with a beam diameter of roughly 80 mm and an on-target power of 100 TW @ 1 Hz were focused by an *f*/30 off-axis parabola mirror onto a gas target, and the vacuum beam radius *w*_0_ was measured to be 32 *u*m at 1/*e*^*2*^, reaching a peak intensity of 3.6 × 10^18^ W/cm^2^ (see Methods). A cascaded LWFA consisting of two-segment pure helium gas jets^6^,^8^ with a special structured gas flow was designed to produce tunable high-quality e-beams for generating tunable narrowband and brilliant γ-rays. The electrons were self-injected in the second bucket of the laser-driven wakefield in the first-segment high-density plasma, and then seeded into the first bucket in the second-segment low-density plasma via rephasing for re-acceleration^26^,^30^. In order to avoid gradient injection in the injector, a steep density bump instead of a downward shock-front was constructed between the high-density injector and the low-density accelerator. The density bump could stop the re-injection of electrons and phase slippage between the plasma wake and the accelerated electrons owing to the increase of the wake phase velocity, producing a high-quality seed e-beam in a quasi-phase-stable way (a separate publication in the future have explained the underlying physics in detail).

**Figure 2:**
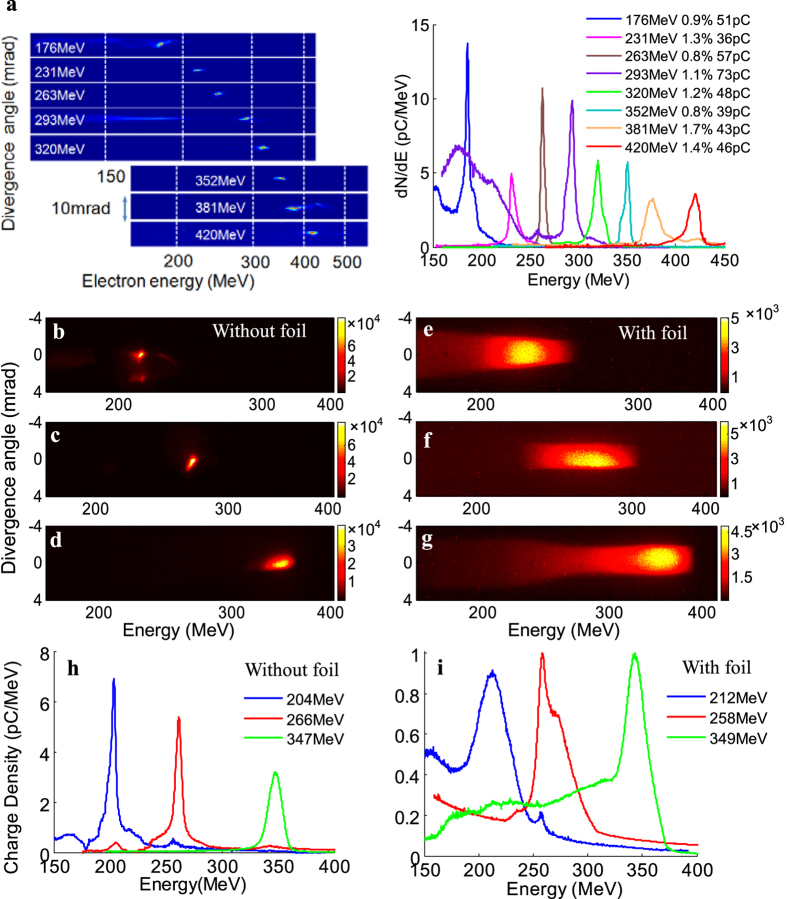
Measured electron spectra without and with the thin foil. (**a**) Angle integrated energy spectra of tunable high-quality e-beams spectra with peak energies in the range of 160 to 420 MeV; (**b–d**) The peak energy, rms energy spread, integrated charge, and rms divergence of the three e-beams are: **(b)** 204 MeV, 1.2%, 52 pC, 0.29 mrad; (**c**) 266 MeV, 1.1%, 46 pC, 0.48 mrad; (**d**) 347 MeV, 1.7%, 45 pC, 0.43 mrad, respectively; (**d–f**) The corresponding spectra of the e-beams after penetrating the thin foil; (**h,i**) Spatially integrated electron spectra for the shots above.

The plasma density and laser focal position were firstly adjusted and optimized to generate reproducible monoenergetic e-beams, then the peak energy of produced e-beam could be manipulated by adjusting the horizontal span between the two gas jets to control the acceleration length in the cascaded LWFA. By adjusting the acceleration length from 1.5 mm to 3 mm, high-quality e-beams with peak energies tunable from 176 to 420 MeV were obtained as shown in Fig. 2a. These e-beams possessed rms energy spread of ~1%, average integrated charge of ~50 pC at peak energy and rms divergence of <0.5 mrad. Under the optimized injection and acceleration conditions, the statistic fluctuation of e-beams’ central positions imaged at 3.6 m away from the source was measured to be less than 2 mm, which means that the high-quality e-beams’ pointing stability was around 0.5 mrad. The peak energy uncertainties due to the e-beams pointing could be less than 1%. Owing to the shot-to-shot fluctuation in laser power within 3% and small jitter in gas density, stable e-beams with fluctuation of ±5% in peak energy were generated and the uncertainty of the measured e-beam charge was estimated within 12%. Since the accelerated e-beam was located just several tens microns (about half of the plasma wavelength) behind the driving laser pulse, the reflected laser pulse while hitting on the foil could inherently overlap the e-beam due to the self-synchronized head-on collision Compton scattering. The driving laser pulse was blocked by the foil but the γ-ray photons could transmit the foil without absorption.

After interaction with the intense reflected laser pulse to generate γ-rays, the disturbed e-beam after penetrating the foil with a heated plasma mirror on the front surface was also analyzed by the 90 cm-long dipole electromagnet spectrometer (see Methods). Different from the undetectable interference on the e-beams by blocking of the 1-mm-thick glass^20^ and the results that the transmitted e-beams were perturbed slightly^24^, it was found that the e-beams’ properties degraded significantly with the energy spread being increased to ~11%, and divergence to ~3.5 mrad as shown in Fig. 2e–g,i, if compared to the e-beams without the foil in Fig. 2b–d,h. However, the beam charge kept almost unchanged. In order to minimize the interference from the bremsstrahlung radiation induced by low-energy large-divergence background electrons (<10 MeV) hitting on the chamber wall or pipelines, a 10-cm-thick wolfram collimator with a 2-mm hole was placed in the chamber to block the peripheral low-energy electrons.

### Numerical results

**Figure 3:**
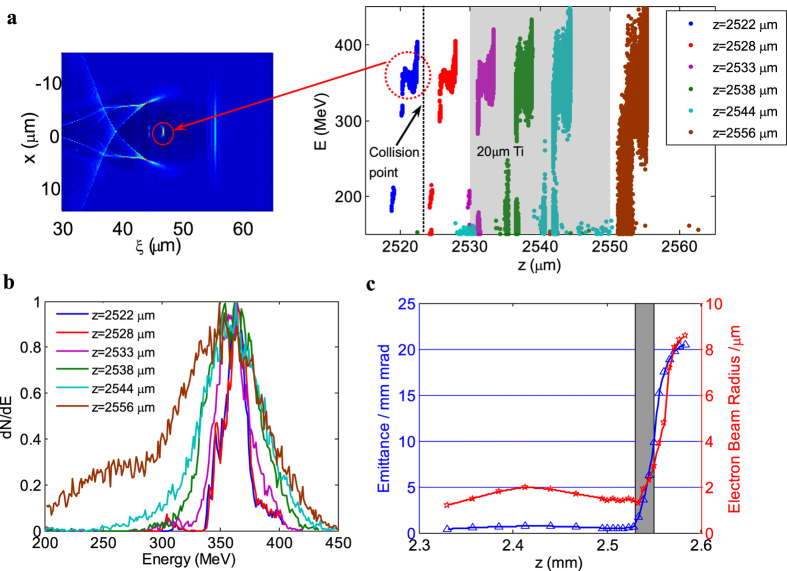
Evolutions of the e-beam in the PIC simulation. (**a**) Snapshot of the two-dimensional electron density distribution and evolution of the electron phase space (*z*, *E*) when penetrating the foil; (**b**) Corresponding e-beam spectra at z = 2.522, 2.528, 2.533, 2.538, 2.5444 and 2.555 mm, respectively; (**c**) Corresponding evolutions of the normalized transverse emittance and the e-beam radius versus the propagation distance z.

Particle-in-cell (PIC) simulations using the Vorpal Code were carried out to get insight on the details of the e-beams generation and degradation. A linearly polarized laser with λ_L_ = 0.8 μm, *a*_0_ = 1.6, *w*_0_ = 32 μm and τ_FWHM_ = 35*f*s was set as the experimental parameters (see Methods). One e-beam peaked at 363 MeV with 1.2% rms energy spread, 0.7 mrad rms divergence, and 26 pC charge was produced and entered into the vacuum region starting from z = 2.49 mm. The 20-um-thick dense plasma with a density of 300 *n*_c_ was placed at z = 2533 um to reflect the driving laser, and head-on collision with the e-beam occurred around z = 2524 um. The e-beam’s properties such as energy spread, emittance remained almost unchanged after the collision with the reflected laser, but began to degrade significantly while penetrating the thin plasma layer, as shown in Fig. 3. It could be explained by that the e-beam was defocused firstly by the self-generated azimuthal magnetic fields along the target front surface^31^, then degraded due to electron-ion collisions inside the foil, as well as the longitudinal and lateral electromagnetic sheath field^32^,^33^, which were built up as the fast electrons reach the rear target surface. However, the degradation of the e-beams properties by the foil did not deteriorate the γ-rays’ generation at all.

### Ultrahigh Brilliance quasi-monochromatic MeV γ-rays

**Figure 4:**
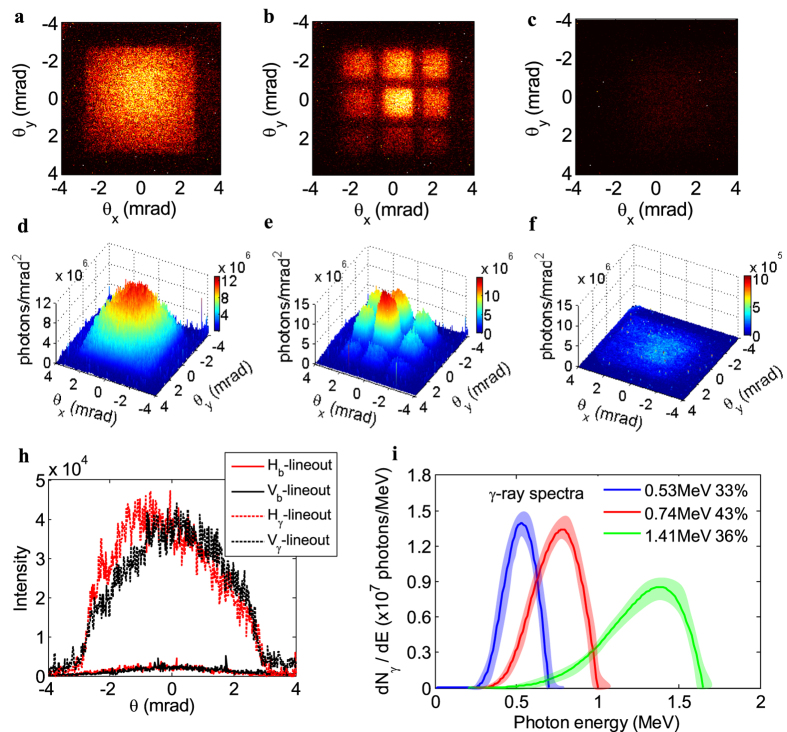
Raw data of γ-ray beam patterns and the reconstructed spectra. (**a**) Typical background-subtracted Gaussian-profile γ-ray beam; (**b**) Imaged γ-ray beam profile transmitted through a 3 × 3 squared lead-grid filter, with the thickness of 1.5, 1, 0.5, 2.5, 0, 2, 5, 4, 3 mm respectively from upper left to low right; (**c**) Imaged background profile when the foil was placed at z = 10 mm; (**d–f**) Corresponding photons distribution on the detected system; (**h**) Horizontal and vertical lineouts of the γ-ray profile and the background profile; (**i**) Reconstructed γ-ray spectra under different e-beam energies in Fig. 2b–d, and the shaded bands represent the spectra uncertainties.

As shown in Fig. 4a–f, the typical γ-ray beam patterns were recorded without and with inserting a lead-grid filter in front of the Lu_2_SiO_4_(LSO)-crystal scintillator (see Methods), where the foil was placed at the exit of the gas flow (z = 0) to obtain the maximum γ-ray photon yield. The Compton scattering γ-ray signal intensity is ~40× higher than the background signal, resulting in a high signal-to-noise ratio Compton source as shown in Fig. 4h. The detected γ-ray beam with a near-Gaussian profile had the FWHM divergence angles of 3.8 and 4.1 mrad in horizontal and vertical directions, respectively. For a head-on collision Compton scattering with a laser pulse at intensity of *a*_*r*_, the number of generated γ-ray photons *N*_p_ per shot can be estimated by^34^, 
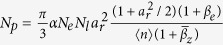
, where, α = e^2^/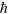
c = 1/137 is the fine structure constant, 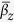 the average axial electron velocity normalized by the light speed, *N*_e_ and *N*_l_ the number of the electrons and laser periods, respectively, and <n> is the average harmonic number, which approaches up to unity in our case. Assuming a nearly head-on collision (using the interaction angle *φ* ≈ 178° to prevent the damage to the laser system by back-propagating light), the radiated photon energy is given by *ħ*ω_γ-ray_ ≈ *ħ*ω_laser_ × 4γ^2^/(1 + *a*_*r*_^2^/2 + γ^2^*θ*_*o*_^2^) eV and at its harmonics, with a typical divergence *θ* ≅ *K*/γ (~1.6 mrad for the 300-MeV e-beam) around the electron velocity vector. *θ*_*o*_ is the observation angle with respect to the electron propagation direction. The laser intensity and off-axis observation can induce a red-shift, resulting in lower photon energies. The total photon number was measured to be of (4.8 ± 0.3) × 10^7^ per shot, which was in a reasonable agreement with the predicted one of ~8 × 10^7^.

In order to identify and evaluate the contribution of the irrelevant radiations to the overall emission, careful measurements have been performed. The betatron radiation from the 360 MeV e-beams in the wakefield had the critical energies *ħ*ω_c_ ≈ 10.5 ± 1 keV either with the single photon counting method or γ-ray detection system (see Supplementary Fig. S1). The keV-energy photon number was measured to be less than 3 × 10^5^, and decreased to ~1 × 10^5^ owing to the absorption of the thin foil. Bremsstrahlung radiation was minimized as possible by using such a thin foil. By carrying out Monte Carlo simulations with the MCNP code, the 50-pC, 300-MeV e-beam would produce less than 3.8 × 10^5^ photons (<3 MeV) within 8 mrad in the forward direction for the 20-um Ti foil, corresponding to 1.1 × 10^−3^ photons for each electron. It indicated that the total photon number from the betatron and bremsstrahlung radiation was nearly two orders of magnitude smaller than the detected γ-ray photon number of ~5 × 10^7^.

**Figure 5:**
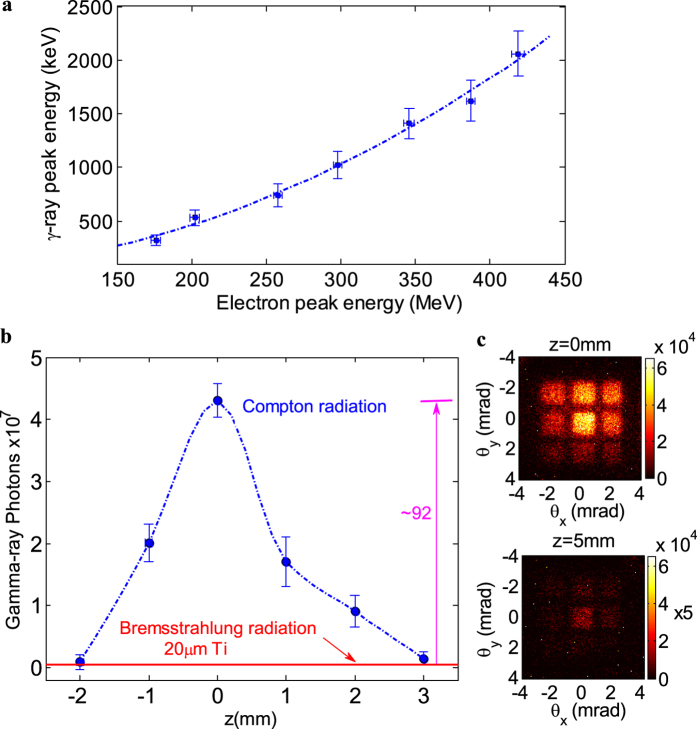
Measured γ-ray energy tuning and detected γ-ray photons as a function of the foil position. (**a**) The measured γ-ray peak energy (each point for the single-shot data), plotted versus with the tunable e-beams peak energy, is fitted with the blue dash line, closed approximately to a 2γ^2^ scaling. The horizontal and vertical error bars correspond to the uncertainties of the e-beams and γ-rays peak energies, respectively; (**b**) Detected γ-ray photons as a function of the foil position. Each data point corresponds to an average over five consecutive shots at different z, and the error bars indicate the fluctuations of γ-ray yield. The bremsstrahlung background at z = 6 mm was plotted as the red line; (**c**) Raw γ-ray beam patterns with a nine-grid filter at z = 0 mm and z = 5 mm, respectively.

The spectra characteristics of Compton scattering can be deduced from indirect filter-pack measurements^20^−^22^,^24^. To measure the photon spectra, a 3 × 3 squared lead-grid filter filled with the thickness from 0.5 mm to 1 cm, or a sixteen-hole-array filter packed with the 1–5.5 cm long attenuated bars made of Al, Cu or W materials (see Supplementary Fig. S2), was placed to attenuate different-energy photons. Then according to the transmission data from each part of the filter, taking the beam profile into account and excluding other irrelevant radiations, the spectra were reconstructed by the expectation-maximization (EM) method based on transmission measurements^35^,^36^ (see Methods). Since the data contained a certain amount of noise from the actual measurement uncertainties and spectra reconstruction resolution, the resulting uncertainties in the peak energies were estimated to be less than 20%. In Fig. 4i, three retrieved spectra of γ-ray beams peaked at 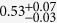, 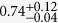 and 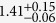 MeV with the corresponding e-beams in Fig. 2b–d, having FWHM bandwidths of 33 ± 5%, 43 ± 6% and 36 ± 4% respectively. By choosing different e-beams energy from 160 to 420 MeV, γ-ray beams peak energy can be tunable from 0.3 MeV up to 2 MeV. As shown in Fig. 5a, the dashed curve was fitted to show the measured γ-ray peak energy plotted versus the measured e-beam peak energy, which was roughly estimated as a 2γ^2^ scaling and as well as close to a more exact fitting scaling given as *ħ*ω_γ-ray_ ≈ *ħ*ω_laser_ × 4γ^2^/(1 + *a*_*r*_^2^/2 + γ^2^*θ*_*o*_^2^) if considering the off-axis observation angle of less than 1 mrad and electron energy in the range from 200–400 MeV.

The γ-ray beam bandwidth was the result from the interplay among the e-beam’s energy spread (~2∆γ/γ), e-beam’s emittance (~γ^2^ϵ^2^), laser bandwidth (~∆ω/ω), laser intensity, and observation angle^37^. The laser propagating in the plasma experienced the blue- and red-shifts arising from the photon acceleration or ionization and local pump depletion^5^,^10^, respectively. The measured transmitted laser spectra still contained most of the unshifted light^6^, but with more red-shifted portions than that of blue-shifted, indicating that the γ-ray spectra would be broadened and shifted to lower energy. Furthermore, the shot-to-shot fluctuation in e-beam pointing on the filters would bring in a detected off-axis angle less than 1.2 mrad to enhance these effects. In the case of the reconstructed spectrum with peak energy of 0.53 MeV and FWHM bandwidth of ~33%, the energy spread (FWHM ∆γ/γ ≈ 3%) and emittance (ϵ ≈ 1.2 mm mrad) of the e-beams contributed only a fractional bandwidth of ~8%. Therefore, the increased bandwidth originated from the convolution of γ-ray spectra reconstruction resolution (~15% at 0.53 MeV) and the others (~25%) from the back-reflected laser, off-axis detection angle, and laser ponderomotive force. A test particle simulation, which calculated the electron trajectories scattered by a transverse Gaussian laser field and the resulting radiation from the Lienard-Wiechert potentials (see Supplementary Fig. S3), had also confirmed these emission spectral broadening and red-shifting phenomena.

The foil position z could play important roles in the γ-ray flux as shown in Fig. 5b. Once the thin foil was hit by the self-focusing laser pulse, it was moved to provide an undamaged surface. While moving the foil inside the gas jet (z < 0), the plasma density profile would be affected, resulting in the interference to electron injection and acceleration in the wake-field, and the γ-ray photon yield diminished as well. When the foil was moved away from the exit of the gas jet (z > 0), the self-focusing laser pulse would diverge as propagating in the vacuum and the laser intensity decreased rapidly at the head-on collision position, leading to the obvious decrease of Compton scattering radiation. The γ-ray photon number was less than 4.5 × 10^5^ while moving the foil 6 mm away from the exit of the gas jet, where bremsstrahlung radiation dominated the overall emission, as consistent with the aforementioned Monte Carlo simulations. The detected γ-ray emission at z = 0 was believed to arise substantially from the electrons wiggling in the laser field. The fluctuation of the e-beams’ directivity and charge led to the fluctuation in γ-ray photon yield. In a series of 100 shots under optimized conditions, the γ-ray beams (>0.3 MeV) can be detected in 28% of these shots.

The generated e- and γ-ray beams parameters are shown in the Table 1. Based on the 2D and 3D PIC simulations, the γ-ray source size and temporal duration which are comparable to the values of the e-beam are estimated as 4 *u*m and 8 *f*s, respectively. These simulated values are also comparable with the experimentally measured values^20^,^38^,^39^. According to the aforementioned γ-ray source experimental parameters, such as photon number (4.8 ± 0.3) × 10^7^, spectrum and beam divergence (~3.8 × 4.1 mrad^2^), we have approximately (7.1 ± 0.5) × 10^4^ photons in a 0.1% bandwidth around the peak energy of 0.74 MeV, giving a peak brilliance of (3.1 ± 0.4) × 10^22^ photons s^−1^ mm^−2^ mrad^−2^ 0.1% BW. This significant increase in the peak brilliance as compared with the previously reported results^20^,^21^,^22^,^23^,^24^,^25^ can mainly be attributed to the high-quality e-beams from the specially designed cascaded LWFA with low energy spread, low emmitance and large charge, and the head-on collision scheme using self-synchronized Compton scattering. To the best of our knowledge, this compact MeV γ-ray source has the peak brilliance one order of magnitude higher than ever reported value of its kinds so far in the literatures. Furthermore, the γ-ray flux of ~5 × 10^7^ photons/shot is comparable to the state-of-the-art Compton scattering sources^40^,^41^,^42^ based on the conventional accelerators and the average brilliance can be improved by several feasible methods in the future, such as by increasing the laser shooting rate and the e-beam charge.

## Discussion

In conclusion, we have demonstrated a self-synchronized all-optical Compton scattering scheme to generate quasi-monochromatic and ultrahigh brilliant MeV γ-rays. With careful design and sophisticated measurements, irrelevant emission including bremsstrahlung and betatron radiations contributed fairly small to the overall γ-ray photons, backed by the exhaustive simulations and the theoretical analysis. Thanks to the tunable high-quality e-beams generated from the specially designed cascade acceleration and the self-synchronized Compton scattering scheme, the produced γ-ray source is tunable from 300 keV up to 2 MeV, possessing high photon number of ~5 × 10^7^ per shot, small divergence of ~4 mrad, narrow bandwidth of ~33% (FWHM) and ultrahigh peak brilliance beyond 10^22^ photons s^−1^ mm^−2^ mrad^−2^ 0.1% BW. The broadened bandwidth of the scattered laser pulse, off-axis detection angle arising from the e-beams’ directivity instability, shot-to-shot fluctuation, as well as the spectral retrieval resolution plays non-ignorable roles in broadening the reconstructed γ-ray spectra. Besides, because the γ-ray spatial distribution or directionality is photon energy dependent, the γ-ray bandwidth in the center of the beam pattern can be narrower. It is worth to note that this self-synchronized all-optical Compton scattering has its own drawbacks. The properties of the scattering laser light cannot be controlled independently and thus it is not flexible to operate in linear scattering regime to achieve minimal x-ray bandwidth. Nevertheless, this efficient and compact ultrahigh brilliance quasi-monochromatic MeV γ-ray source, based on the perfect combination of a laser-plasma accelerator and a plasma mirror, has its own advantages as aforementioned, which may be of interest to provide a few practical and experimental applications in the future, such as nuclear resonance fluorescence, x-ray radiology and ultrafast pump-probe nondestructive inspection.

## Methods

### Laser and target for cascaded LWFA

The experiments were carried out at the femtosecond 200 TW laser system^29^ based on the chirped-pulse amplification (CPA) Ti:sapphire with a 1-Hz repetition rate. The 33*-f*s, 800-nm laser pulses with a beam diameter of roughly 80 mm and an on-target power of 100 TW were focused by an *f*/30 off-axis parabola mirror into the gas jet and the vacuum beam radius *w*_0_ was measured to be 32 *u*m at 1/*e*^2^, the peak intensity was estimated to be 3.6 × 10^18^ W/cm^2^, corresponding to a normalized amplitude of *a*_0_ = 1.3. The fractional laser energy contained within the laser spot was measured to be ~59%. The gas target consisted of two-segment pure (0.8 mm + 3 mm) helium gas jets, which were used to produce a structured gas flow for realizing cascaded acceleration. A probe beam split from the main laser beam was sent perpendicularly across the gas jet, then entered a 4*f* Michelson-type interferometer for measuring the plasma density. The first-segment gas jet was filled with pure He atoms with an ionized electron background density of (1.1 ± 0.1) × 10^19^ cm^−3^ along the laser axis propagation and the second-segment gas flow was operated with an average plasma density of (6.5 ± 0.5) × 10^18^ cm^−3^ as measured via the optical interferometry. A steep density bump instead of a downward shock-front was constructed between the high-density injector and the low-density accelerator.

### Electron beam measurements

The laser-accelerated e-beams were deflected by a 90-cm-long tunable dipole electromagnet (3.6 m after the exit of the gas jet) with a maximum magnetic field of 1.1 Tesla, corresponding to an energy resolution of 0.06% at 300 MeV with 0.1 mrad divergence, and measured by a Lanex phosphor screen (PS) imaged onto an intensified charge-couple device 16-bit (ICCD) in a single shot, which was cross-calibrated by using a calibrated imaging plate to measure the charge of the e-beams^43^. Before presenting the foil, the electrons’ single-shot far field distribution, directivity and divergence were analyzed and optimized.

### Simulations

The e-beam generation in the underdense plasma and degradation when crossing the foil (regarded as a dense plasma) were all performed using Vorpal, a fully electromagnetic PIC code. The linearly polarized laser pulse with wavelength λ_0_ = 800 nm, normalized laser peak amplitude *a*_0_ = 1.6, duration 33*f*s FWHM and spatial intensity profile was taken close to the experimental parameters. To reduce the simulation time, a moving window with a size of 75 × 160 μm^2^ was used and moved at the speed of light. The simulation grid size was k_0_x = 0.087 in the laser propagation direction and k_0_z = 0.175 in the transverse direction with four macroparticles per cell. The longitudinal plasma profile consisted of an 192 μm-long upward density ramp followed by a 0.6-mm-long plateau with a density of 1.1 × 10^19^ cm^−3^, and then a 160 μm-long downward density ramp from the maximum density of 1.6 × 10^19^ cm^−3^ to 0.9 × 10^19^ cm^−3^, which was followed by a 1.5-mm-long slow downward plasma with a average density of 0.6 × 10^19^ cm^−3^. The e-beam then interacted with the 20 um-thick dense plasma and its evolution was recorded until z = 2.9 mm.

### γ-ray beam diagnostic

Aiming at the expected parameters of the resulting γ-rays, a measurement system, compound with the radiology imaging technique, was proposed, consisting of a filter, a scintillating crystal, a CCD camera and a master computer. The Lu_2_SiO_4_(LSO)-crystal scintillator with a diameter of 50 mm was placed downstream, 5.2 m away from the exit, to induce fluorescence by the generated Compton γ-ray beams within a collecting solid angle of 9.6 mrad, which was recorded by an imaging CCD camera. The γ-ray detection system was encased by the lead baffles to minimize the background noise and a signal to noise ratio of 30 dB could be obtained. It was calibrated using a Co^60^ (1.25 MeV) radiation source of known activity, taking into account the intrinsic detector response including quantum efficiency as well as the transmitted materials. Based on the calibrated γ-rays detected system, the total photon number could be measured precisely within the scope of 3% error.

### Spectrum Analysis

A filter cut into a 3 × 3 square lead-grid with each of 7 × 7 mm^2^ or a sixteen-hole-array with each of 5 mm diameter, filled with different thickness of absorbed material, was aligned finely in front of the LSO-crystal scintillator. When the photon fluence *F* was casted on a detector, the signal measured could be expressed as 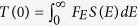, where 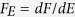 was the derivative energy spectrum, *S*(*E*) the signal produced by unit fluence of photon. Once a filter with the thickness of *x* was placed in the beam path, the photon spectrum was changed to 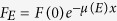 and the signal *T* became 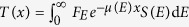, where *μ*(*E*) was the filter’s attenuation coefficient. Assuming the total energy zone was divided into *n*-1 intervals with each of 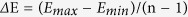, the discrete equations for the intensity signal of the *i*^th^ attenuator could be displayed as 
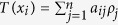
, where 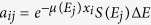, 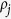
(j = 1, 2, …, n) the intensity in the *j*^th^ energy zone. Since a series of attenuators were employed, corresponding equations can be formulated to reconstruct the task γ-ray spectrum. The unfolding algorithm of Expectation Maximization Method^35^ was developed and improved to solve the combined linear equations. In the procedure of reconstruction, the expected energy zone is divided into 100 bins. Due to this improved algorithm and convergent conditions, the spectra could be reconstructed smoothly by increasing and optimizing the iteration times. This sophisticated technique has been tested to work well in our previously-designed experiments, which has been carried out on Philips X’Unique II X-ray source in the 0~3 MeV range with suitable filters. It has also a good agreement with the spectra simulated by Monte Carlo method.

Furthermore, due to the uneven spatial distributions of γ-rays irradiated on the attenuator area, a correction factor *p* indicating the distribution of γ-ray beam should be taken into account, leading the aforementioned equation to be 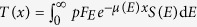. The spatial distribution *p* of emergent gamma-rays could be gained through maximum likelihood estimation of the data measured with the detection system, consisting of only scintillator and image receiving equipment (without the filter), under a consecutive run of ~ 50 shots for Compton scattering emission.

## Additional Information

**How to cite this article**: Yu, C. *et al*. Ultrahigh brilliance quasi-monochromatic MeV γ-rays based on self-synchronized all-optical Compton scattering. *Sci. Rep.* **6**, 29518; doi: 10.1038/srep29518 (2016).

## Supplementary Material

Supplementary Information

## Figures and Tables

**Table 1:** Typical experimental parameters for e-beams and γ-ray beams.

*Electron beam*	
Tunable peak energy	160–420 MeV
Energy spread	0.8–1.5% (rms)
Total charge	30–120 pC
Bunch diameter (*sim*.)	3 μm
Duration (*sim.*)	6 *f*s
Divergence	0.2–0.5 mrad (rms)
*Ray source*	
Tunable peak energy	0.3–2 MeV
Bandwidth	33–60% (FWHM)
Photons/shot	~5 × 10^7^
Source size (*sim.*)	4 μm
Divergence	4 mrad (FWHM)
Peak brilliance†	~3 × 10^22^

^†^In units of photons s^−1^ mm^−2^ mrad^−2^ per 0.1% bandwidth.
