# Supplementary material for: Ultrahigh brilliance quasi-monochromatic MeV γ-rays based on self-synchronized all-optical Compton scattering
Source: Sci Rep. 2016 Jul 13;6:29518. doi: 10.1038/srep29518 (PMC4942800; doi:10.1038/srep29518)
Supplement: Supplementary Information [file srep29518-s1.pdf]

Ultrahigh brilliance quasi-monochromatic MeV γ-rays based on self-synchronized all-optical Compton scattering

Changhai Yu[1], Rong Qi[1], Wentao Wang[1], Jiansheng Liu[1, 2, *],Wentao Li[1], Cheng Wang[1], Zhijun Zhang[1], Jiaqi Liu[1], Zhiyong Qin[1], Ming Fang[1], Ke Feng[1], Ying Wu[1], Ye Tian [1], Yi Xu[1], Fenxiang Wu[1], Yuxin Leng[1], Xiufeng Weng[3], Jihu Wang[3], Fuli Wei[3], Yicheng Yi[3],Zhaohui Song[3], Ruxin Li[1,#], Zhizhan Xu[1,†]

[1]*State Key Laboratory of High Field Laser Physics, Shanghai Institute of Optics and Fine Mechanics, Chinese Academy of Sciences, Shanghai 201800, China*

[2]*IFSA Collaborative Innovation Center, Shanghai Jiao Tong University, Shanghai 200240, China*

[3] *State Key Laboratory of Intense Pulsed Radiation Simulation and Effect, Northwest Institute of Nuclear Technology, Xi'an 710024, China*

*michaeljs_liu@siom.ac.cn,

#ruxinli@mail.shcnc.ac.cn,

†zzxu@mail.shcnc.ac.cn

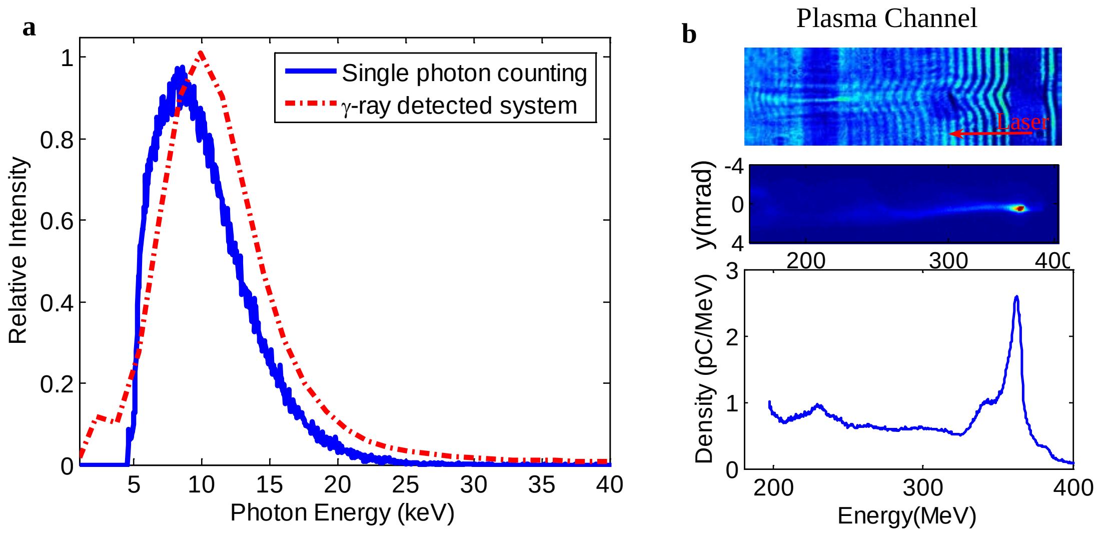

Supplementary Figure 1 | The betatron x-ray diagnostic and generated e-beam. (a) Single-shot x-ray betatron spectra obtained from the single-photon counting method (SPC) method (blue), detected by a thermoelectrically cooled, back-illuminated, deep-depletion x-ray CCD cameras and the residual laser light was blocked by a 80nm-thick Al filter placed in front of the x-ray CCD, comparing with the γ-ray detected system (red), both measured with the critical energy around $\hbar\omega_c \approx 5.24\times 10^{-24}\ \gamma^2\ n_e[\mathrm{cm}^{-3}]\ r_\beta[\mu\mathrm{m}]$ keV $\approx(10.5\pm1)$ keV; **(b)** The measured plasma channel via the optical interferometry and the corresponding e-beam spectra: peak energy 360MeV, 1.4% rms energy spread, 49.7pC integrated charge, 0.35mrad rms divergence.

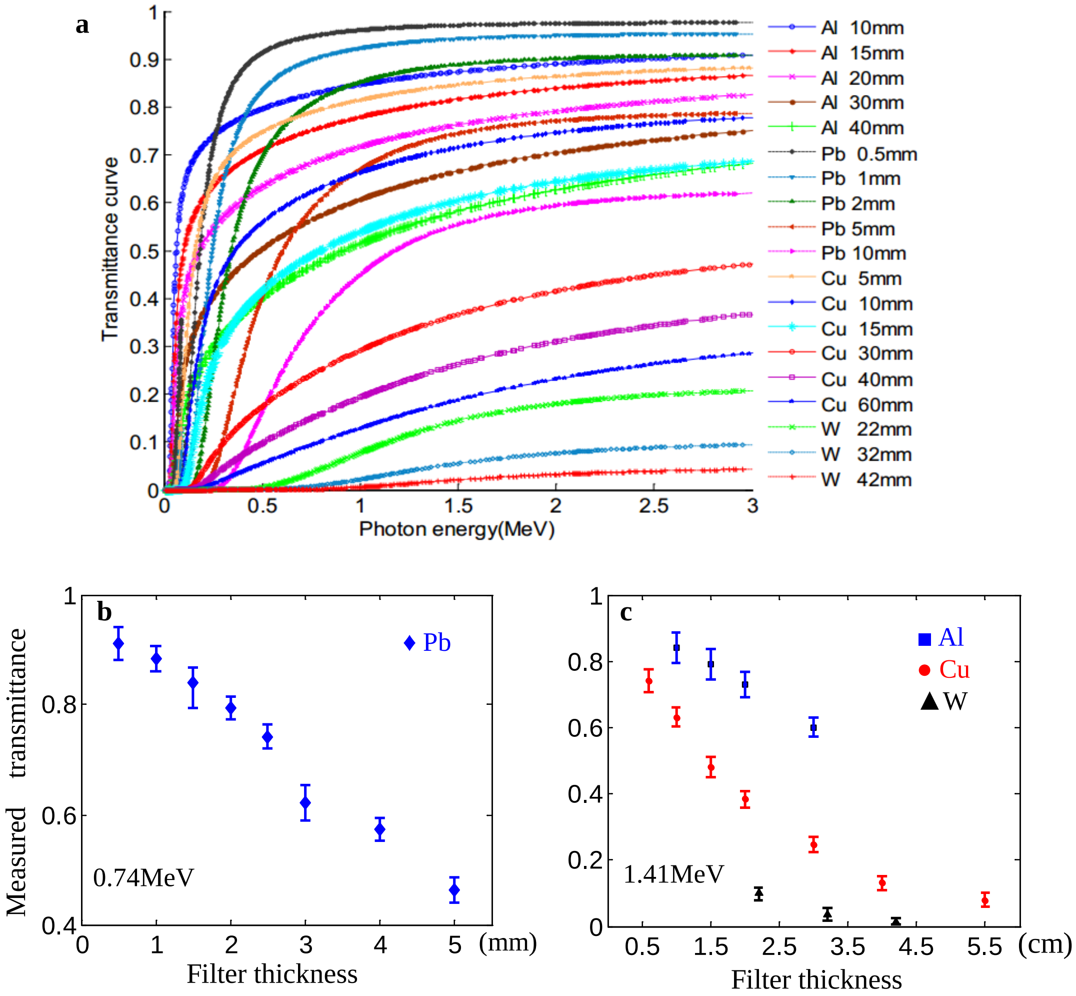

Supplementary Figure 2 | The material transmittance and measured γ-ray signal including angular correction vs. filter thickness. (a) Material transmittance of the filters with different thickness for Al (10-40mm), Pb (0.5-10mm), Cu (5-60mm), W (22-42mm); (b-c) Typical transmission data for reconstructing the typical spectra with peak energies at 0.74MeV and 1.41MeV, using a 3×3 squared lead-grid (♦) filter and a sixteen-hole-array filter packed with Al (■), Cu (●) and W (▲) material attenuated bars, respectively. The error bars of each point represents the measurement uncertainties considering the beam spatial profile angular correction.

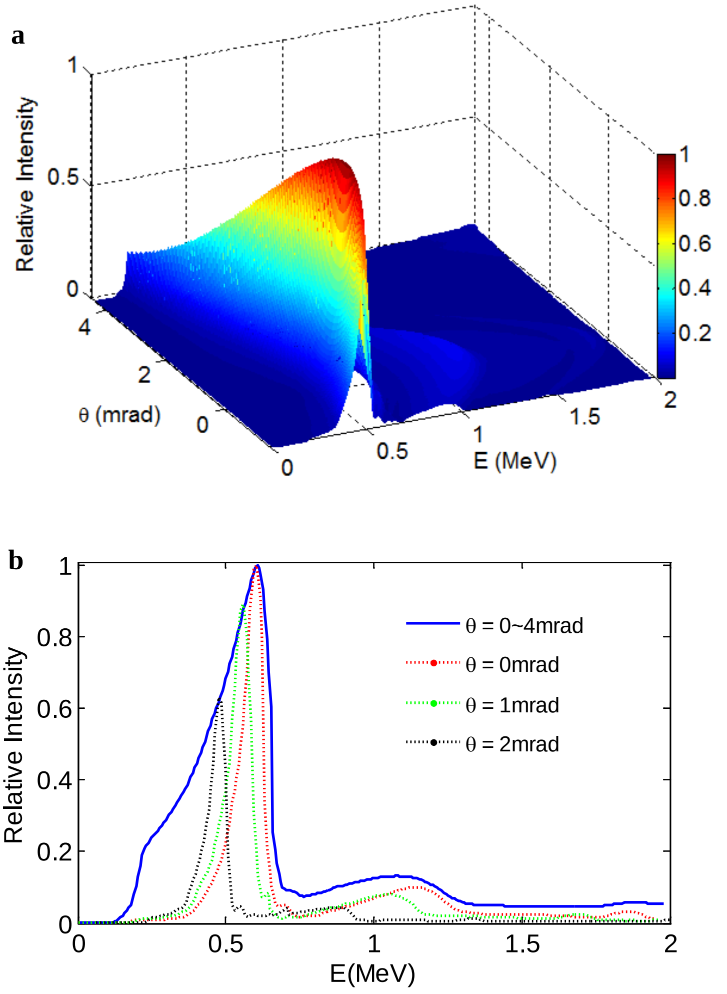

Supplementary Figure 3 | Spectra radiation simulations. (a) The 10000 test particles are used to sample an collimating 200 MeV electron beam with 3% FWHM energy spread. Relative radiation intensity distribution for the electrons wiggling in the intense laser field N_l=13, a_r=0.95 based on the direct numerical integration of the Lienard-Wiechert potentials in the far field; **(b)** The angular-resolved radiation intensities integrated with respect to the emission angles from 0 to 4mrad, the center axis θ = 0 mrad, and off-axis angle detected angle θ =1mrad and θ =2mrad, respectively.
